# Acylated Ghrelin Receptor Agonist HM01 Decreases Lean Body and Muscle Mass, but Unacylated Ghrelin Protects against Redox-Dependent Sarcopenia

**DOI:** 10.3390/antiox11122358

**Published:** 2022-11-28

**Authors:** Rojina Ranjit, Holly Van Remmen, Bumsoo Ahn

**Affiliations:** 1 Department of Biochemistry, University of Oklahoma Health Science Center, Oklahoma City, OK 73104, USA; 2 Aging and Metabolism Research Program, Oklahoma Medical Research Foundation, Oklahoma City, OK 73104, USA; 3 Oklahoma City VA Medical Center, Oklahoma City, OK 73104, USA; 4 Gerontology and Geriatrics, Internal Medicine, Wake Forest University, Winston-Salem, NC 27106, USA

**Keywords:** unacylated ghrelin, HM01, skeletal muscle, oxidative stress, sarcopenia, muscle weakness

## Abstract

Sarcopenia, the progressive loss of muscle mass and dysfunction, universally affects the elderly and is closely associated with frailty and reduced quality of life. Despite the inevitable consequences of sarcopenia and its relevance to healthspan, no pharmacological therapies are currently available. Ghrelin is a gut-released hormone that increases appetite and body weight upon acylation, which activates its receptor GHSR1a. Recent studies have demonstrated that acyl and unacylated ghrelin are protective against acute pathological conditions of skeletal muscle. We hypothesized that both acyl ghrelin receptor agonist (HM01) and unacylated ghrelin ameliorate muscle atrophy and contractile dysfunction in oxidative stress-induced sarcopenia. HM01, unacylated ghrelin, or saline was delivered via osmotic pump. HM01 increased food consumption transiently, while the body weight remained elevated. It also decreased lean body mass and muscle mass of wildtype and Sod1KO. In contrast, unacylated ghrelin ameliorated loss of muscle mass by 15–30% in Sod1KO mice without changes in food consumption or body weights. Contractile force was decreased by ~30% in Sod1KO mice, but unacylated ghrelin prevented the force deficit by ~80%. We identified downregulation of transcription factor FoxO3a and its downstream E3 ligase MuRF1 by unacylated ghrelin. Our data show a direct role of unacylated ghrelin in redox-dependent sarcopenia independent of changes of food consumption or body weight.

## 1. Introduction

The proportion of the world’s population over 60 years of age will nearly double from 12% to 22% between 2015 and 2050 according to the prediction by World Health Organization. One of the consequences of aging is the progressive loss of muscle mass and strength (i.e., sarcopenia). Sarcopenia often leads to a substantial loss of mobility and independence, affecting healthy life span in the elderly in the absence of diseases [1,2]. The rapid growth in geriatric population and the significance of sarcopenia in life quality calls for attention for sarcopenia interventions. However, pharmacological therapies effective for sarcopenia are lacking. 

Ghrelin is a hormone with 28-amino acid sequence, which is produced predominantly from the P/D1 cells in the stomach [3]. A subset of ghrelin undergoes acylation at the serine 3 site via ghrelin O acyl transferase. Upon translational modification, acyl ghrelin binds to the receptors in the hypothalamus and pituitary glands, growth hormone secretagogue receptor 1a (GHSR1a) [4]. The receptor activation increases appetite, alters metabolic rates, and leads to increased growth hormone production and activation of insulin like growth factor-1 (IGF-1) signaling [3,4]. The circulating level of acyl ghrelin changes throughout the day in response to low energy state [3,4]. A previous study showed that administration of ghrelin and genetic deletion of ghrelin both, paradoxically, prevent aging associated muscle atrophy and dysfunction [5]. In addition to the GHSR1a receptor activation, acyl and unacylated ghrelin have direct impacts on peripheral tissues, including skeletal muscle [6,7,8]. Thus, it is important to determine the effects driven by receptor activation of ghrelin in sarcopenia. HM01 is a recently characterized non-peptidic agonist of GHSR1a receptor that increases food intake and body weights [9,10,11]. This small molecule compound also conferred protections against muscle wasting in tumor-bearing rodents [9,10]. HM01 has a higher binding affinity and longer plasma half-life than acyl ghrelin [11]. 

While a small subset (~5–10%) of ghrelin is acylated depending on energy status, the majority of ghrelin in the circulation is unacylated ghrelin [7]. Biological effects of unacylated ghrelin have been demonstrated in multiple tissues, including skeletal muscle. Graziani group [7] demonstrated that exogenous administration of unacylated ghrelin increases myofiber cross-sectional area in fasting- and denervation-induced muscle wasting, while downregulation of proteolytic pathways is involved in muscle protein degradation, including FoxO3a, the ubiquitin proteasome system and autophagy pathways. Unacylated ghrelin also increases satellite cell function and damage-induced myofiber regeneration [6,12], which is impaired in advanced age. In skeletal muscle wasting elicited by chronic kidney diseases, unacylated ghrelin attenuated muscle wasting and mitochondrial oxidative stress and respiration [13]. Further, unacylated ghrelin protects against mitochondrial defects and redox imbalance in muscle [13,14] and mitochondrial-targeted apoptosis in neurons [15], demonstrating its effects on multiple tissues involved in sarcopenia. Thus, unacylated ghrelin may protect against sarcopenia where neurogenic atrophy and oxidative stress play significant roles. 

Mice lacking cytosolic superoxide dismutase, Sod1KO, have been established as a mouse model of redox dependent sarcopenia. The Sod1KO mice exhibit high levels of oxidative stress, mitochondrial dysfunction and oxidative damage associated with impairment of neuromuscular junction, mimicking underlying causes of sarcopenia in humans [16,17,18,19,20] and other mammals [21,22,23,24]. These mice show significant loss of muscle mass beginning at two months of age [25], but they do not exhibit significant behavioral or other physiological alterations that affect skeletal muscle health, including inactivity, decreases in food consumption, or hormonal changes [21,22]. 

Here, we asked whether GHSR1a receptor agonist HM01 and unacylated ghrelin prevent the onset of loss of muscle mass and weakness in a model of redox-dependent sarcopenia. HM01 induced loss of muscle mass in wildtype mice and exacerbated sarcopenia in Sod1KO. However, unacylated ghrelin increased muscle mass and improved contractile properties in Sod1KO mice without altering food consumption or body mass. Our results further demonstrate that unacylated ghrelin is associated with downregulation of FoxO3a and its downstream pathway E3 ligases. Our results demonstrate that unacylated ghrelin protects against loss of muscle mass and function in redox dependent sarcopenia, which warrants further investigation in age-associated muscle atrophy and weakness.

## 2. Materials and Methods

### 2.1. Animal Care

C57Bl/6 wildtype and Sod1KO female mice used in the study were generated by Dr. Charles Epstein’s laboratory and have been previously described [26,27]. All mice were caged in a pathogen free environment with free access to standard chow and water and maintained on a 12 h light/dark cycle. We established that the Sod1KO mice exhibit an early onset of sarcopenia characterized by atrophy, contractile dysfunction, mitochondrial ROS, and neuromuscular disruption [21,23,28], which are typical phenotypes of sarcopenia in humans [17,19,29]. Wildtype and Sod1KO mice were treated with HM01, unacylated ghrelin or saline at two months of age for one or two months, the age prior to the onset of redox-dependent sarcopenia. The Institutional Animal Care and Use Committee at Oklahoma Medical Research Foundation approved all procedures. 

### 2.2. HM01 and Unacylated Ghrelin Delivery

Alzet Osmotic minipumps (1.5 cm long × 0.6 cm diameter, 0.4 g weight; #1004; Durect Corporation, Cupertino, CA, USA) were filled and equilibrated with sterile saline 24 h prior to implantation. Mice were anesthetized using isoflurane. We shaved and disinfected the skin over implantation site. A 1 cm incision was made through the skin on the back, and space was made for the pumps by blunt dissection with sterile forceps. Upon insertion of a pump, skin was sutured with sterile absorbable ETHIILON 6.0 sutures (ETHICON, San Lorenzo, Puerto Rico). Minipumps were then inserted subcutaneously under sterile conditions. Mice received constant release of HM01 (10 mg/kg/day, Helsinn Healthcare SA, Switzerland), unacylated ghrelin (100 ug/kg/day, Anaspec^®^, Fremont, CA, USA) or saline for one or two months. Concentrations of each treatment is based on their protective effects in vivo from previous publications [7,10]. Mice were kept on electric heating pads throughout the procedure and until complete recovery from anesthesia when they were returned to their home cage. Daily food consumption was measured as the difference in weight between the food put into the cage and that remaining at the end of 24 h. Daily food consumption is then normalized by the animals’ body weight. 

### 2.3. Assessment of Body Composition 

Quantitative magnetic resonance imaging (qMRI) was used to determine the percentages of body fat and lean mass [30]. Awake mice were placed into a thin-walled plastic cylinder (4.7 cm ID, 0.15 cm thick), where they were free to turn around but were limited to ∼4 cm vertical movements by a plastic insert. The plastic cylinder containing the live mice was then placed into the qMRI machine (EchoMRI, Echo Medical Systems, Houston, TX, USA) for measurement of lean and fat mass. qMRI measurements of body fat and lean mass provide increased precision, accuracy, speed of results, and ease of use compared with dual-energy X-ray absorptiometry or chemical methods. Once the measurements were completed, mice were returned to their home cage. 

### 2.4. In Vitro Contractile Properties and Force Generation 

Studies of limb muscle isometric contractile properties were assessed using extensor digitorum longus (EDL) in vitro as previously described [31]. Mice were sacrificed using gaseous carbon dioxide, and EDL muscle was immediately excised and prepared for functional assays in a bicarbonate-buffered solution gassed with a mixture of 95% O_2_ and 5% CO_2_ at room temperature. We placed the EDL muscle in an organ bath containing bicarbonate-buffered solution at room temperature. We adjusted muscle length to attain maximal twitch tension (optimal length, Lo), increased the temperature of the organ bath to 37 °C, and after thermo-equilibration (20 min) started our force-frequency protocol. The isometric force-frequency protocol consisted of pulse frequencies of 1–300 Hz interspersed by 1 min intervals. To estimate the bundle CSA, we divided the EDL muscle weight (g) by length (cm) multiplied by muscle specific density (1.056) based on previous established equation [32]. Isometric force data were normalized by estimated muscle cross-sectional area (CSA; N/cm^2^) for calculation of specific force. All data were recorded and analyzed using commercial software (DMC and DMA, Aurora Scientific, Aurora, Canada). 

### 2.5. Western Blot

Tissue lysates from gastrocnemius were used to determine expressions of specific proteins. Snap frozen gastrocnemius tissues (~20 mg) were homogenized in Ripa buffer, containing 50 mM Tris-Cl (pH 7.4), 1 mM EDTA, 0.5 mM EGTA, 1% Triton X-100, 0.1% sodium deoxycholate, 0.1% SDS, 140 mM NaCl. Equal amounts (10–20 µg) of protein samples were loaded on 10–12.5% SDS PAGE gels (casting system from Bio-Rad Laboratories, Hercules, CA, USA) with 1x Tris/Glycine/SDS buffer. Gels were transferred to nitrocellulose or PVDF membranes using wet transfer system (Bio-Rad Laboratories) overnight at 16-18 V in Tris/Glycine/SDS buffer containing 15% methanol then blocked for one hour in 1% bovine serum albumin (BSA) in TBS buffer containing 0.1% Tween-20 (TBS-T). The membranes were incubated with primary antibodies overnight, followed by 1 hr incubation with secondary antibodies. The blots were imaged using Odyssey DLx imaging system and quantified by Image StudioLite^®^ and Empiria Studio^®^ softwares (LI-COR, Lincoln, NE, USA). Signal intensities were normalized by total proteins stained by Ponceau or Revert 700. We used the following primary antibodies: FoxO3a, Santa Cruz Biotechnology (Santa Cruz, CA, USA), SC-48348; phospho-FoxO3a (Ser253), Cell Signaling Technology (Danvers, MA, USA), 9466S; MuRF1, Santa Cruz, sc-398608; Pax 7, Santa Cruz, sc-81648; MyoD, Santa Cruz, sc-377460; ribosomal protein S6, Santa Cruz, sc-74459; phospho-S6 ribosomal protein (Ser240/244), Cell Signaling, 2215. 

### 2.6. Statistical Analysis 

Graphpad Prism 9 (GraphPad Software, San Diego, CA, USA) was used for statistical analyses. Two-way ANOVA was used to determine main effects by genotype and treatment as well as the interaction effects. These tests were followed by Tukey post hoc tests as indicated by individual figures. Data are expressed as means ± SEM. Statistical significance was set at *p* < 0.05. 

## 3. Results

### 3.1. HM01 Treatment Transiently Increases Food Intake, but Leads to Decreases in Lean Body Mass and Muscle Quantity

**Figure 1:**
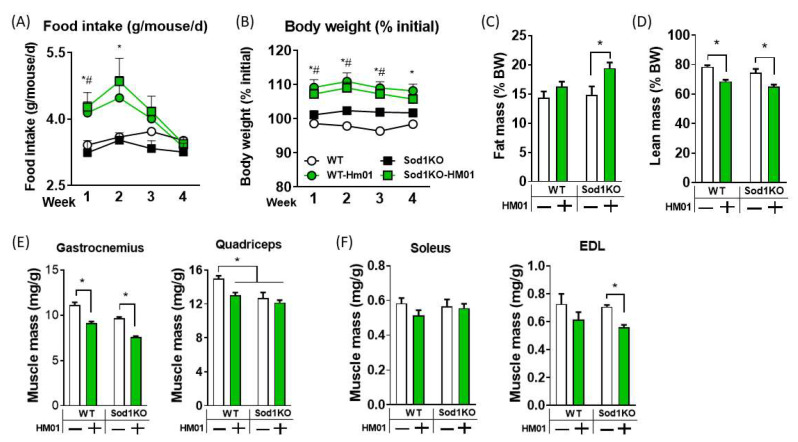
Four-week treatment of HM01 increases food intake transiently but decreases lean body mass and muscle mass. (**A**) Food consumption during the four-week HM01 treatment. (**B**) Percent body weight relative to initial over four-week study period. (**C**,**D**). % fat mass and % lean mass relative to body weight at study endpoint. (**E**) Gastrocnemius and quadriceps normalized to body weight. (**F**) Soleus and extensor digitorum longus normalized to body weight. Two-Way ANOVAs were used to test statistical significance between groups, followed by Tukey post hoc tests. Data are mean ± SEM. *p* < 0.05. * significant difference between WT and WT-HM01. # Significant difference between Sod1KO and Sod1KO-HM01. Abbreviation: +, treated; −, untreated; EDL, extensor digitorum longus.

Sod1KO mice maintain body weight similar to wildtype animals until two months of age, and the mice lose muscle mass at 3 months of age, which then gradually exacerbates over time [25]. Therefore, we treated two months old wildtype and Sod1KO mice with HM01 for one month prior to onset of sarcopenia. Mean initial body weights of individual groups were ~18–20 g (Table S1A). We found that HM01 significantly increased food intake by ~30% for both wildtype and Sod1KO mice in the first two weeks, but the orexigenic effect rapidly diminished in the final two weeks and dropped to wildtype level (Figure 1A). Body mass was increased in HM01 treated mice and remained elevated until the study endpoint unlike food consumption data (Figure 1B). Percent fat mass was ~5% increased by HM01 in Sod1KO mice (Figure 1C). Percent lean body mass was decreased by ~15–20% by HM01 in wildtype and Sod1KO mice (Figure 1D). Consistent with the loss in percent lean body mass, skeletal muscle masses were also decreased by HM01 treatment in wildtype and exacerbated in fast twitch muscles of Sod1KO mice (Figure 1E,F). HM01 treated group increased body weight, but muscle mass failed to increase accordingly (Figure S1A). Mass of soleus muscle did not differ by genotype or HM01 treatment (Figure 1F).

### 3.2. Longer Term Treatment of HM01 Results in Loss of Muscle Mass and Reduced Maximum Force Generation

**Figure 2:**
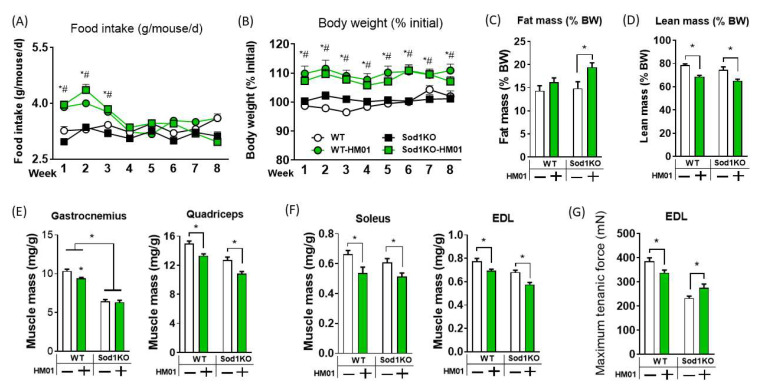
Mice treated with HM01 for eight weeks exhibit loss of muscle mass and reduced maximum force generation. (**A**) Food intake during the eight-week HM01 treatment. * significant difference between WT and WT-HM01. # significant difference between Sod1KO and Sod1KO-HM01. (**B**) Percent body weight relative to initial over eight-week study period. (**C**,**D**) Percent fat mass and percent lean mass relative to body weight at the study endpoint. (**E**) Gastrocnemius and quadriceps normalized to body weight. (**F**) Soleus and extensor digitorum longus (EDL) normalized to body weight. (**G**) Maximum tetanic force generation of EDL muscle at 200 Hz electrical stimulation. Data are mean ± SEM. Two-Way ANOVAs were used to test statistical significance between groups, followed by Tukey post hoc tests. Data are mean ± SEM. * *p* < 0.05.

One of the possibilities for the lack of protection against sarcopenia is the treatment period. Thus, we treated wildtype and Sod1KO mice for eight weeks. Mean initial body weights of individual groups were ~20–24 g (Table S1B). During the eight-week treatment, we observed transient orexigenic effect and persisting increases in body mass throughout the study period consistent with our 4 weeks treatment (Figure 2A,B). HM01 increased percent fat mass in Sod1KO, while the trend of increase in wildtype did not reach statistical significance (Figure 2C). HM01 again decreased the percent lean body mass in wildtype and Sod1KO mice consistent with the four-week treatment study (Figure 2D). Gastrocnemius muscle mass was decreased by HM01 in wildtype mice, but no changes were observed in Sod1KO mice (Figure 2D; Figure S1B). HM01 decreased quadriceps muscle mass in wildtype and Sod1KO mice (Figure 2E; Figure S1B). The representative slow twitch muscle mass (i.e., soleus) was significantly decreased by HM01 treatment (Figure 2F), which was not the case in four-week treatment study. Finally, both EDL muscle mass and maximum tetanic force generation were reduced showing decreased quality and quantity by HM01 (Figure 2F,G) in wildtype mice. In contrast to the wildtype mice, maximum tetanic force was increased in Sod1KO mice. We anticipate that HM01 may have protective effects on impairment in excitation-coupling in Sod1KO mice. For example, increased calcium kinetics (release and uptake) or increased calcium sensitivity by sarcomeric proteins can improve tetanic force in Sod1KO mice. We have reported impaired calcium handling in Sod1KO previously [33,34].

### 3.3. GHSR1a Receptor Downstream Pathways and Mechanisms Are Involved in Myogenesis and Protein Synthesis in Muscle

**Figure 3:**
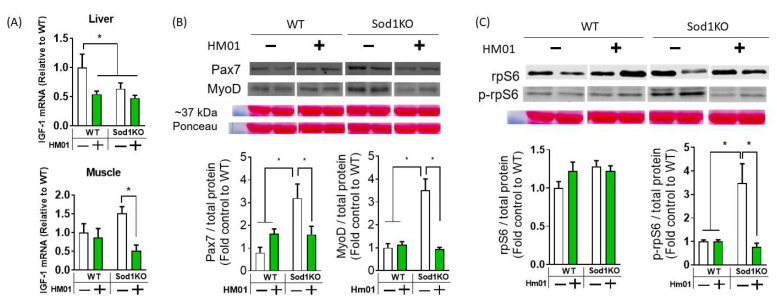
Four-week HM01 treatment abrogates anabolic pathways in Sod1KO. (**A**) IGF-1 levels in the liver and muscle are significantly decreased after HM01 treatment. (**B**) Proteins involved in myogenesis, Pax7 and MyoD, are upregulated in Sod1KO muscle. This upregulation is abrogated by HM01 treated Sod1KO group. (**C**) Ribosomal protein S6 levels were unchanged, but its phosphorylation is significantly upregulated at Ser240/244 sites. HM01 treated mice are lacking this upregulation of the marker for ribosomal protein synthesis. Two-Way ANOVAs were used to test statistical significance between groups, followed by Tukey post hoc tests. Data are mean ± SEM. * *p* < 0.05.

To further understand the effect of HM01 on GHSR1a downstream pathways, we determined IGF1 levels in the liver and muscle, both of which were downregulated (Figure 3A,B). We found that Pax 7 and MyoD were upregulated in Sod1KO which is presumably a compensatory response to muscle wasting. However, this was abrogated by HM01 (Figure 3B). Consistent with these findings, ribosomal protein synthesis marker S6 was downregulated by HM01 (Figure 3C). These reductions in anabolic pathways and protein synthesis may have contributed to the exacerbation of muscle loss in Sod1KO. HM01 resulted in loss of muscle mass in wildtype mice and exacerbated in the animal model of redox dependent sarcopenia. These findings are associated with downregulation of IGF1 pathway and myogenesis and protein synthesis in muscle.

### 3.4. Unacylated Ghrelin Increases Muscle Mass without Changes in Food Consumption or Body Weights

**Figure 4:**
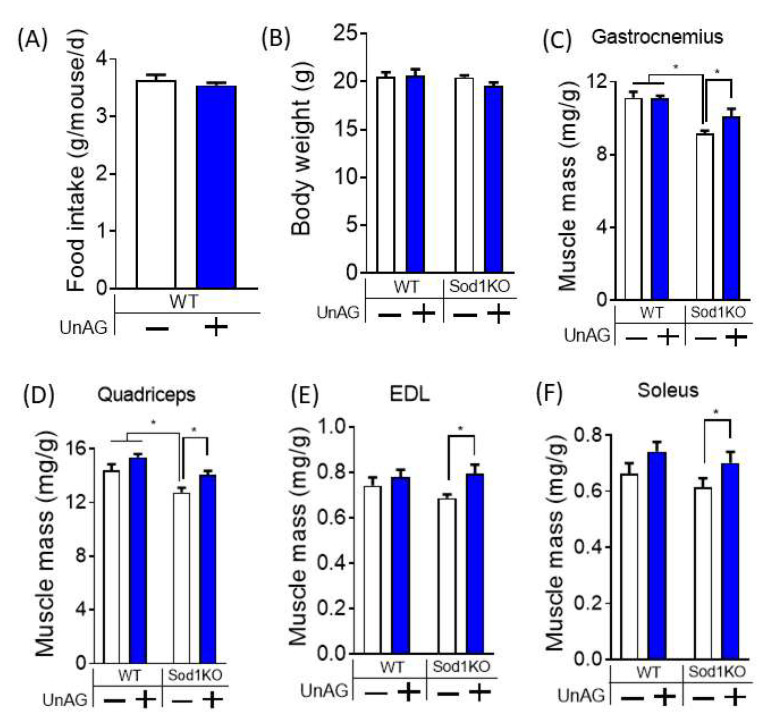
Unacylated ghrelin treatment increases skeletal muscle mass in redox-dependent sarcopenia independent of changes in food consumption or body weight. Unacylated ghrelin does not alter food intake (**A**) or body (**B**) of wildtype mice. (**C**,**D**) Unacylated ghrelin preserves the loss of muscle mass of gastrocnemius and quadriceps in Sod1KO mice. (**E**,**F**) Unacylated ghrelin increases muscle mass of EDL and soleus in Sod1KO mice. Two-Way ANOVAs were used to test statistical significance between groups, followed by Tukey post hoc tests. Data are mean ± SEM. * *p* < 0.05. Abbreviations: UnAG, unacylated ghrelin; EDL, extensor digitorum longus.

We tested direct effects of ghrelin in skeletal muscle in vivo without its activation on GHSR1a receptors. Two months old Sod1KO mice were treated with unacylated ghrelin for 4 weeks. Mean initial body weights of individual groups were ~18–20 g (Table S1C). Unacylated ghrelin increased weights of gastrocnemius and quadriceps by ~20–30% in Sod1KO mice (Figure 4C,D and Figure S1C). We also found increased masses of slow twitch and fast twitch muscles of Sod1KO mice, including EDL and soleus (Figure 4E,F). Unacylated ghrelin did not alter food intake or body weight of the mice (Figure 4A,B). These findings indicate that the protective effects of unacylated ghrelin against redox dependent sarcopenia was independent of food consumption or body weight.

### 3.5. Unacylated Ghrelin Improves Contractile Properties 

**Figure 5:**
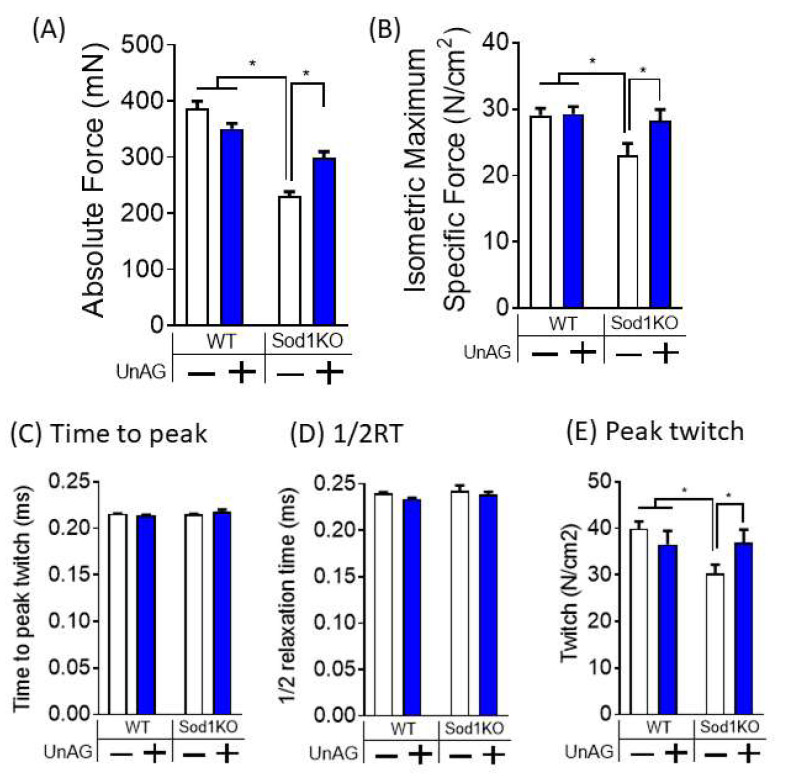
Unacylated ghrelin improves contractile properties of EDL muscle. (**A**) Isometric maximum specific force, force per cross-sectional area, were significantly decreased in Sod1KO, but fully protected by unacylated ghrelin treatment. (**B**) Maximum absolute force generation is significantly lower in Sod1KO mice, but partially protected by unacylated ghrelin. (**C**) Time to reach peak twitch is unchanged by Sod1KO or unacylated ghrelin treatment. (**D**) Half relaxation time (1/2 RT) did not differ by Sod1 deletion or unacylated ghrelin. (**E**) Peak twitch is significantly reduced in Sod1KO but preserved by unacylated ghrelin treatment. Two-Way ANOVAs were used to test statistical significance between groups, followed by Tukey post hoc tests. Data are mean ±SEM. * *p* < 0.05. Abbreviations: UnAG, unacylated ghrelin; EDL, extensor digitorum longus.

To test the effect of unacylated ghrelin in force generation, we assessed in vitro contractile properties of isolated EDL muscle, a representative fast twitch muscle that undergoes preferential effect by aging. We found that maximum tetanic force was decreased by ~50% in Sod1KO mice, but unacylated ghrelin treatment protected against the force deficit by ~50% (Figure 5A). Furthermore, maximum tetanic specific force, force per cross-sectional area, was fully protected by unacylated ghrelin (Figure 5B). This data shows that the impact of unacylated ghrelin in force modulation was greater than changes in muscle mass. To further test the effect of unacylated ghrelin in calcium transient and uptake, we analyzed twitch characteristics of EDL. We found that time to peak twitch and half-relaxation time were unchanged by unacylated ghrelin (Figure 5C,D). However, peak twitch was decreased in muscle from Sod1KO mice, which was increased by unacylated ghrelin (Figure 5E). Collectively, our findings demonstrate that unacylated ghrelin improves muscle quality in Sod1KO mice.

### 3.6. Downstream Pathways Activated by Unacylated Ghrelin 

**Figure 6:**
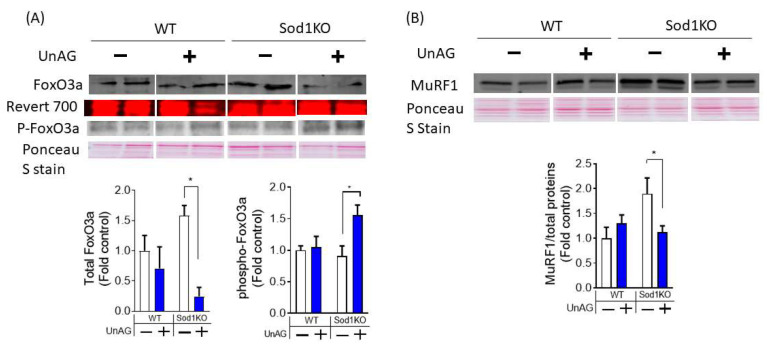
Unacylated ghrelin’s downstream effects in a redox dependent sarcopenia. (**A**) FoxO3a protein expression is decreased in by unacylated ghrelin in Sod1KO muscle. Phosphorylation of FoxO3a at Ser 253 was upregulated by unacylated ghrelin treatment in Sod1KO. (**B**) E3 ligase MuRF1 expression is increased in Sod1KO but downregulated by unacylated ghrelin. Two-Way ANOVAs were used to test statistical significance between groups, followed by Tukey post hoc tests. Data are mean ± SEM. * *p* < 0.05. Abbreviations: UnAG, unacylated ghrelin; FoxO3a, Forkhead box O3a.

Previous studies reported direct effects of unacylated ghrelin in cultured C2C12 myotubes and in muscle tissues after in vivo treatment [6,7]. In cultured myotubes, unacylated ghrelin has been shown to downregulate FoxO3a and its downstream targets. We found that FoxO3a level was decreased, which was associated with increased phosphorylation at Ser 253 site (Figure 6A). We further observed upregulation E3 ligase MuRF1 in Sod1KO, which was normalized by UnAG (Figure 6B), suggesting the effects of unacylated ghrelin on proteolytic pathways in Sod1KO mice. 

## 4. Discussion

The goal of our study is to determine the receptor-dependent and -independent effects of ghrelin in redox dependent loss of muscle mass and weakness. We first tested the hypothesis that GHSR1a receptor activation via HM01 prevents loss of muscle mass and contractile function in Sod1KO mice. GHSR1a receptor agonist HM01 induced loss of muscle mass in wildtype mice and exacerbated redox-dependent sarcopenia. However, treatment with unacylated ghrelin, the form that does not bind to GHSR1a receptor, substantially improved muscle mass and contractile properties in Sod1KO mice. Our results collectively demonstrated that unacylated ghrelin is protective against the loss of muscle mass and function in early onset of sarcopenia, while HM01 induces adipogenesis and accelerates loss of lean body mass and muscle mass. 

GHSR1a activation by HM01 increased appetite in wildtype animals as anticipated, but food intake was elevated only for the first two weeks. Two recent studies also have demonstrated an increase in cumulative food intake by HM01 treatment for two weeks [9,10]. The increase in body weight was associated with adiogenic effect of HM01 in our study, which is consistent with previous findings [9,10]. Here, we further demonstrate that the orexigenic effect of HM01 disappears after the initial two weeks. Regardless of the transient impact of appetite, the body weight remained elevated throughout the study period. The maintained body weight throughout the study period might be related to the effect of GHSR1a activation on metabolism and a reduction in activity, as previous studies have shown decreased resting energy expenditure by HM01 [10]. 

HM01 decreased percent lean body mass and muscle mass, in contrast to increases in lean body mass and gastrocnemius muscle mass in other papers [9,10]. The reason for the discrepancy is unclear, but it might be due to the treatment duration, type (oral gavage vs. osmotic pump), pharmacokinetic exposure profile, drug dose or different animal species and models (sarcopenic Sod1KO vs. cachectic tumor bearing animals) [9,10]. Our findings show that 4-8 weeks of continuous activation of GHSR1a receptor using HM01 reduces percent lean body mass and limb muscle mass, which is primarily driven by increases in body mass. We also find that HM01’s wasting effect becomes more prominent with prolonged treatment because as muscle tissues are affected longer by two months treatment (Figure 1, Figure 2 and Figure S1). The mechanisms of this time-dependent effect of HM01 is currently unknown. It is noteworthy that muscle groups responded differently after HM01 treatment, where one month treatment reduced weights of muscle groups primarily composed of fast twitch fibers while soleus weight remained unchanged (Figure 1). We anticipate that the heterogeneity of fiber composition plays an important role in this differential impact. For example, age-associated loss of muscle mass has been shown to affect fast and slow twitch fibers differentially in humans and rodents. It is also possible that the density of ghrelin receptors in muscle membranes might be also different depending on fiber types. The peptide sequence of ghrelin receptors in muscle are currently unknown, so the receptor density cannot be determined based on our current knowledge [31,35,36]. 

HM01 exacerbated sarcopenia in our mouse model of redox-dependent sarcopenia. Consistent with the data in wildtype mice, food consumption and body weight effects were also observed in redox-dependent sarcopenia. Decreased muscle mass in Sod1KO was exacerbated by HM01. We found that IGF-1 mRNA levels in the liver and in gastrocnemius muscle were downregulated, suggesting suppression of GHSR1a downstream, growth hormone and IGF-1 pathways. The proteins involved in myogenesis (i.e., Pax 7 and MyoD) were upregulated in Sod1KO muscle, which is presumably the compensatory response to muscle wasting. However, HM01 abrogated this upregulation, which might have contributed to worsening of sarcopenia in Sod1KO. These findings are inconsistent with the previous evidence showing the activation of IGF-1 signaling pathways by GSHR1a activation [7]. We predict that the longer-term treatment contributes to the discrepancy. It is interesting that specific force, force generation per area, in Sod1KO was increased by HM01 treatment regardless of muscle loss. We anticipate that HM01 may have protective effects on impairment in excitation-coupling in Sod1KO mice. For example, increased calcium kinetics (release and uptake) or increased calcium sensitivity by sarcomeric proteins can improve tetanic force in Sod1KO mice. We have reported impaired calcium handling in Sod1KO previously [33,34]. 

The wasting and weakness by HM01 led us to test the direct impact of ghrelin on peripheral tissues. We used unacylated ghrelin to test ghrelin’s direct effects independent of the GHSR1a receptor activation. The direct impacts of both types of ghrelin were widely reported in multiple peripheral tissues, including skeletal muscle, bone, neurons [37,38]. Unacylated ghrelin increased muscle mass and contractile function in Sod1KO mice. Consistent with these findings, studies have shown that both acylated and unacylated ghrelin increased myotube proliferation and differentiation demonstrating its direct impacts [6,7]. Several studies in vivo demonstrated unacylated ghrelin’s protective effect against acute wasting conditions, including fasting, denervation, burn-injury, chronic kidney diseases, and high-fat diet induced wasting [7,13,39,40]. The level of protection against wasting in our mice was ~20–30% in gastrocnemius and quadriceps, but. This level of protection is consistent with another study against denervation and fasting-induced atrophy [7]. 

Unacylated ghrelin protects against impaired contractile function of redox dependent sarcopenia. Notably, unacylated ghrelin fully restored muscle force generation in Sod1KO mice, effect that was greater for muscle quality than muscle quantity. The mechanisms by which unacylated ghrelin increases force is a subject for future investigations, but it might involve improvements in calcium kinetics. Neuroprotective effect of unacylated ghrelin was abrogated by L-type calcium channel blocker in cultured neuronal cells [41], suggesting the role of calcium release mechanisms. Likewise, decrease in peak twitch tension in Sod1KO, indirect assessment of calcium release and sensitivity, was recovered by unacylated ghrelin. It would be interesting to directly measure the effect of unacylated ghrelin in calcium release and sensitivity in future studies. These are critical for excitation-contraction coupling of skeletal muscle and known to be impaired in age-associated sarcopenia [42,43].

Previous studies have demonstrated direct effects of unacylated ghrelin in cultured myotubes and in muscle tissues after in vivo treatment. Unacylated ghrelin promoted proliferation and myotube fusion in C2C12 myotubes [6]. Another group demonstrated that the effects by unacylated ghrelin was dependent on FoxO3a, mTORC2 and Akt pathways [7]. Consistent with these data, we found that unacylated ghrelin led to an increase in FoxO3a phosphorylation at Ser 253, which leads to degradation of FoxO3a transcription factor. We indeed observed downregulation of FoxO3a expression in Sod1KO treated with unacylated ghrelin. We further observed that one of the FoxO3a downstream transcription factors, MuRF1, was reduced by unacylated ghrelin. Our results suggest that downregulation of protein degradation pathways, at least in part, contributes to the preservation of muscle mass. Future studies using transcriptomics and proteomics can further elucidate other downstream pathways and molecules affected by unacylated ghrelin. 

## 5. Conclusions

Our data demonstrate that unacylated ghrelin protects against muscle wasting and weakness in redox-dependent sarcopenia. These results warrant future investigations on the effect of unacylated ghrelin in age-associated sarcopenia. Mechanistic studies related to the balance of protein degradation and synthesis and calcium kinetics and sensitivities of sarcomeric proteins will improve our understanding of unacylated ghrelin in sarcopenia. As pointed out by other investigators, the receptor for unacylated ghrelin remains unknown [7,44]. Discovery of the receptor identity can open new opportunities of sarcopenia research and ghrelin biology. We also report transient effect of HM01 in food consumption and reduced lean body mass and muscle mass. 

## Data Availability

Not applicable.

## Supplementary Materials

The following supporting information can be downloaded at: https://www.mdpi.com/article/10.3390/antiox11122358/s1, Figure S1: Skeletal muscle mass in mg tissue weights for HM01 (A,B) and unacylated ghrelin treated mice (C); Table S1: Initial body weights for both HM01 and unacylated ghrelin treated animals.
