# Supplementary material for: Acylated Ghrelin Receptor Agonist HM01 Decreases Lean Body and Muscle Mass, but Unacylated Ghrelin Protects against Redox-Dependent Sarcopenia
Source: Antioxidants (Basel). 2022 Nov 28;11(12):2358. doi: 10.3390/antiox11122358 (PMC9774605; doi:10.3390/antiox11122358)
Supplement: Supplementary file 1 [file antioxidants-11-02358-s001.zip › antioxidants-1925469-supplementary.pdf]

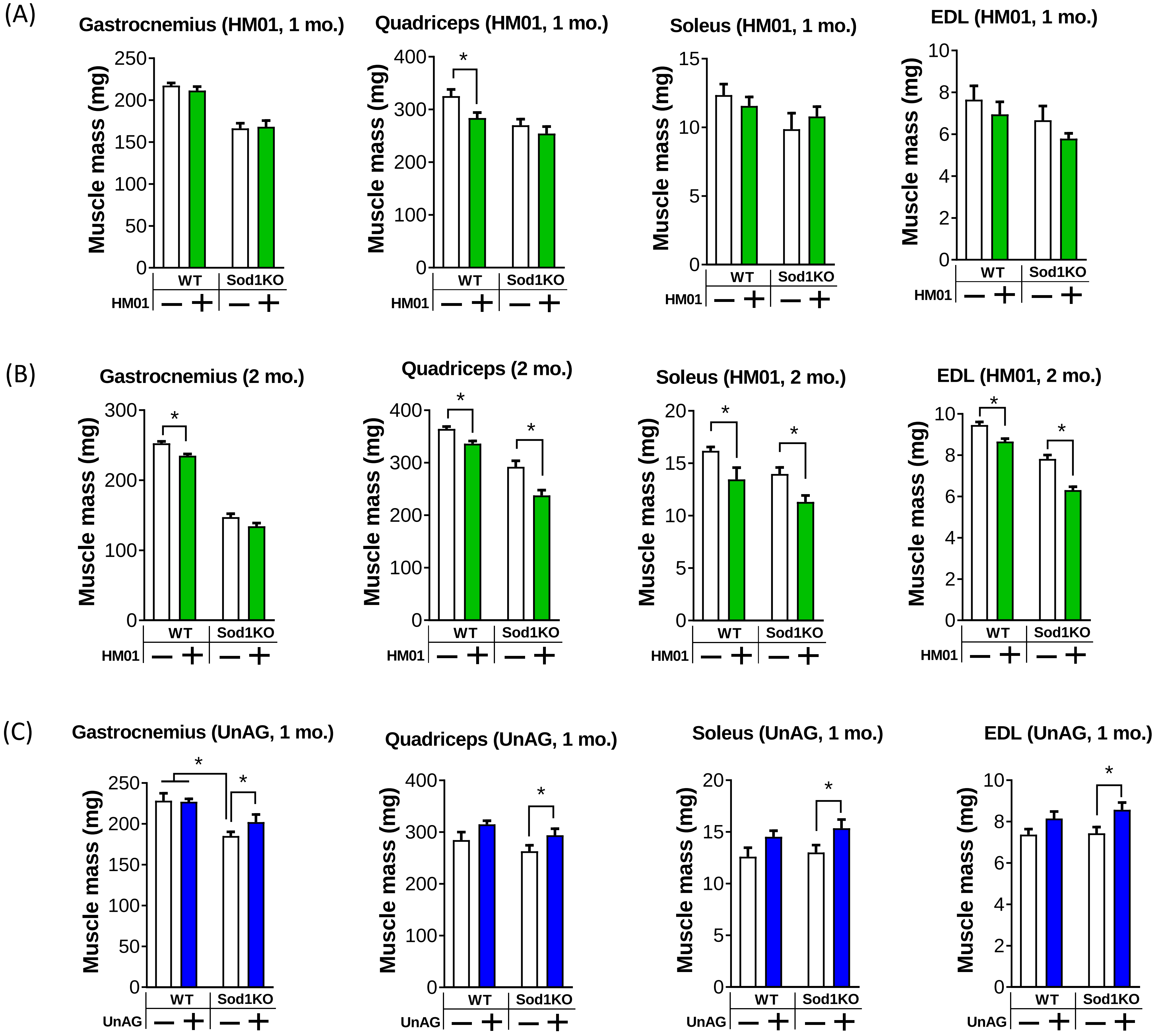

Figure S1. Skeletal muscle mass in mg tissue weights for HM01 (A, B) and unacylated ghrelin treated mice (C).

(A) HM01, 1mo

	WT	WT-HM01	Sod1KO	Sod1KO-HM01
Mean	20.44	18.72	18.77	18.43
SEM	1.64	0.68	2.85	1.28

No statistical differences were detected among groups.

(B) HM01, 2mo

	WT*	WT-HM01	Sod1KO	Sod1KO-HM01
Mean	24.58	22.63	22.00	20.37
SEM	1.42	2.45	1.59	1.10

* WT was significantly different compared to Sod1KO and Sod1KO-HM01.

(C) Unacylated ghrelin, 1 mo

	WT	WT-UnAG	Sod1KO	Sod1KO-UnAG
Mean	20.51	20.45	20.61	18.11
SEM	1.23	0.56	1.97	1.19

No statistical differences were detected among groups.

Supplemental Table S1. Initial body weights for both HM01 and unacylated ghrelin treated animals.
